# Redox Status, Procoagulant Activity, and Metabolome of Fresh Frozen Plasma in Glucose 6-Phosphate Dehydrogenase Deficiency

**DOI:** 10.3389/fmed.2018.00016

**Published:** 2018-02-05

**Authors:** Vassilis L. Tzounakas, Federica Gevi, Hara T. Georgatzakou, Lello Zolla, Issidora S. Papassideri, Anastasios G. Kriebardis, Sara Rinalducci, Marianna H. Antonelou

**Affiliations:** ^1^Department of Biology, School of Science, National and Kapodistrian University of Athens, Athens, Greece; ^2^Department of Ecological and Biological Sciences, University of Tuscia, Viterbo, Italy; ^3^Department of Science and Technology for Agriculture, Forestry, Nature and Energy, University of Tuscia, Viterbo, Italy; ^4^Department of Medical Laboratories, Faculty of Health and Caring Professions, Technological and Educational Institute of Athens, Athens, Greece

**Keywords:** transfusion medicine, fresh frozen plasma, G6PD^−^ donors, donor variation, metabolomics, extracellular vesicles, antioxidant capacity, interactome

## Abstract

**Objective:**

Transfusion of fresh frozen plasma (FFP) helps in maintaining the coagulation parameters in patients with acquired multiple coagulation factor deficiencies and severe bleeding. However, along with coagulation factors and procoagulant extracellular vesicles (EVs), numerous bioactive and probably donor-related factors (metabolites, oxidized components, etc.) are also carried to the recipient. The X-linked glucose 6-phosphate dehydrogenase deficiency (G6PD^−^), the most common human enzyme genetic defect, mainly affects males. By undermining the redox metabolism, the G6PD^−^ cells are susceptible to the deleterious effects of oxidants. Considering the preferential transfusion of FFP from male donors, this study aimed at the assessment of FFP units derived from G6PD^−^ males compared with control, to show whether they are comparable at physiological, metabolic and redox homeostasis levels.

**Methods:**

The quality of *n* = 12 G6PD^−^ and control FFP units was tested after 12 months of storage, by using hemolysis, redox, and procoagulant activity-targeted biochemical assays, flow cytometry for EV enumeration and phenotyping, untargeted metabolomics, in addition to statistical and bioinformatics tools.

**Results:**

Higher procoagulant activity, phosphatidylserine positive EVs, RBC-vesiculation, and antioxidant capacity but lower oxidative modifications in lipids and proteins were detected in G6PD^−^ FFP compared with controls. The FFP EVs varied in number, cell origin, and lipid/protein composition. Pathway analysis highlighted the riboflavin, purine, and glycerolipid/glycerophospholipid metabolisms as the most altered pathways with high impact in G6PD^−^. Multivariate and univariate analysis of FFP metabolomes showed excess of diacylglycerols, glycerophosphoinositol, aconitate, and ornithine but a deficiency in riboflavin, flavin mononucleotide, adenine, and arginine, among others, levels in G6PD^−^ FFPs compared with control.

**Conclusion:**

Our results point toward a different redox, lipid metabolism, and EV profile in the G6PD^−^ FFP units. Certain FFP-needed patients may be at greatest benefit of receiving FFP intrinsically endowed by both procoagulant and antioxidant activities. However, the clinical outcome of G6PD^−^ FFP transfusion would likely be affected by various other factors, including the signaling potential of the differentially expressed metabolites and EVs, the degree of G6PD^−^, the redox status in the recipient, the amount of FFP units transfused, and probably, the storage interval of the FFP, which deserve further investigation by future studies.

## Introduction

Fresh frozen plasma (FFP) is commonly used in transfusion therapy to maintain the coagulation status in patients with acquired multiple coagulation factor deficiencies and severe bleeding after injury (1). In addition, FFP units can be used as a pool for biopharmaceutical fractionation in order to manufacture medicinal products (2). Practically, FFP is used for its ability to generate thrombin and form a clot as a result of intrinsic components, including coagulation factors, calcium and procoagulant phospholipid surfaces, involved in the assembly of coagulation complexes, and coagulation activation (3). However, apart from coagulation factors and procoagulant extracellular vesicles (EVs), numerous bioactive signaling factors and oxidized lipids and proteins (4) pass to the FFP recipient.

The extent of this risk is partly related to inter-individual donor characteristics. Indeed, several studies have recently reported significant donor-to-donor variation in numerous blood properties *in vivo* and in labile blood products. In the case of red-cell concentrates, apart from in-bag hemolysis and 24-h posttransfusion recovery, units from different donors might have substantial variation in antioxidant capacity (5, 6), cellular fragility (6, 7), or surface removal signals (8). Extracellularly, the uric-acid-dependent antioxidant capacity of the supernatant (that influence the storage lesion, and thus, the quality of the blood component) significantly varies among donors (9–11). In the same context, and since the plasma reflects the physiological state of donor’s cells and tissues (12), significant variation has been observed among FFP units used for transfusion, in terms of EV characteristics and lipid peroxidation (4, 13).

Certain aspects of the so-called “donor variation effect” are attributed to genetic factors that dictate subclinical inter-donor differences in blood physiology as clearly exemplified by the distinct blood profile of beta thalassemia trait and glucose-6-phosphate dehydrogenase (G6PD)-deficient donors (14). In the last case, the subjects are characterized by extremely low levels of G6PD activity that catalyzes the first reaction in the pentose phosphate pathway converting glucose 6-phosphate to gluconolactone-6-phosphate. Pentose phosphate pathway feeds cells with reducing equivalents (like nicotinamide dinucleotide hydrogen phosphate, NADPH) needed for the maintenance of redox equilibrium. In cases of oxidative stress, NADPH helps in the regeneration of reduced glutathione, in the detoxification of hydrogen peroxide and in the prevention of oxidative damage in membrane lipids and proteins. G6PD deficiency (G6PD^−^) affects the energy and redox status of cells and consequently, a range of energy-dependent cellular activities, including the transport properties of cell membrane, a feature that might link changes in cell metabolomes to those of plasma (15).

Genetic factors may determine the quality of stored blood and probably, its posttransfusion performance and effects. Thus, a study of donors carrying the most common human enzyme genetic defect might be highly relevant to blood transfusion. Moreover, since G6PD^−^ is an X-linked defect, males are more commonly affected than females. Considering that G6PD activity influences both the cellular and plasma homeostases and that a typical transfusion practice is the use of FFP units donated exclusively by male donors, the study of G6PD^−^ male donors is especially relevant to FFP transfusion. However, and despite this intrinsic clinical interest, little is known about the physiological properties and the metabolome of FFP donated by eligible, G6PD^−^ donors. This study aimed at the comparative assessment of FFP units produced by whole blood donations from G6PD-deficient and -sufficient male donors, by using a number of biochemical measurements, flow cytometry and mass spectrometry, in addition to statistical and bioinformatics tools.

## Materials and Methods

### Blood Donors and Fresh Frozen Plasma (FFP) Preparation

Blood from 12 eligible male regular donors was used for the production of FFP units. G6PD^−^ donors under study (*n* = 6) carried the common Mediterranean variant of G6PD^−^ (16). After donation of approximately 465 mL of blood and addition of 63 mL of CPDA-1 (citrate–phosphate–dextrose–adenine) anticoagulant, clinical-grade FFP was prepared according to the standard blood banking procedures (17), directly from whole blood units at 4°C. Briefly, after centrifugation at 4,500× *g* for 15 min, the supernatant plasma was squeezed off by a plasma expressor (Fenwall Laboratories, Deerfield, IL, USA) and frozen for 12 months at −20°C. For analysis, FFP samples were rapidly thawed for 15–20 min at 30–37°C to avoid precipitation of cold-precipitating proteins, consistent with the blood banking procedure for the thawing of clinical FFP for transfusion and the standard AABB operating procedures. The study was approved by the Ethics Committee of the Department of Biology, School of Science, NKUA. Investigations were carried out upon signing of written consent, in accordance with the principles of the Declaration of Helsinki.

### Free Hemoglobin, Redox Parameters, and Protein Analysis

Free hemoglobin was calculated by using the Harboe method as previously described (10). Total (TAC) and uric-acid-dependent antioxidant capacity (UA/AC) of FFP samples were determined in the absence or presence of uricase (Sigma-Aldrich, Munich, Germany) treatment, respectively (18), by using the ferric reducing antioxidant power assay (19). Lipid peroxidation of FFP units was assessed by measuring the levels of malondialdehyde (MDA), a natural by-product of lipid peroxidation. Briefly, after deproteinization of each sample with 15% trichloroacetic acid, thiobarbituric acid was added (all chemicals by Sigma-Aldrich, Munich, Germany). After heating of the samples for 50 min at 95°C, the absorption of the produced chromogenic MDA–thiobarbituric acid complex was measured at 532 nm. Measurements were plotted against a standard curve of known MDA concentration.

For the FFP protein characterization, 20 µg of FFP samples were separated in homogeneous 10% sodium dodecyl sulfate polyacrylamide gels, transferred onto nitrocellulose membranes, and probed with primary antibodies against advanced glycated end products (AGEs, 1:1,000 Millipore AB9890), soluble clusterin (sCLU, 1:1,000 Santa Cruz Biotechnology), human hemoglobin (Hb, 1:15,000, Europa Bioproducts), IgGs (1:1,000; Sigma I-2011), and horseradish peroxidase-conjugated secondary antibodies. Immunoblots were developed using a standard enhanced chemiluminescence reagent kit and the relative amount of each protein was quantified by scanning densitometry (Gel Analyzer v.1.0 image-processing program, Athens, Greece). In addition, FFP samples were processed for the detection of protein carbonylation using the Oxyblot detection kit as per manufacturer’s specifications (20).

### Extracellular Vesicles (EV) Profiling

Extracellular vesicle-associated procoagulant activity was estimated by using a functional Elisa assay kit (Zymuphen MP-activity, Hyphen BioMed, Neuville-sur-Oise, France) as per manufacturer’s instructions. All FFP samples were supplemented with calcium, Factor Xa, and thrombin inhibitors before addition into microplate wells precoated with streptavidin and biotinylated Annexin V (AnnV). Subsequently, samples were incubated at 37°C before introduction of factor Xa–Va and prothrombin. After addition of the chromogenic substrate, thrombin activation induced by the AnnV positivity (AnnV^+^) EVs was detected at 405 nm and expressed as nM of phosphatidylserine (PS) equivalents.

Enumeration and phenotyping of FFP EVs was performed by flow cytometry within 15 min from units’ thawing, as previously described (13). Briefly, EVs were identified by size (<1 μm), exposure of cell-specific markers, and AnnV^+^. All samples were double stained with AnnV-phycoerythrin (PE Annexin V Apoptosis Detection Kit I, 559763) and CD235a-fluorescein isothiocyanate (clone GA-R2, HIR2, 559943) or integrin-α2b-FITC (CD41a, clone HIP8, 555466) or CD45-fluorescein isothiocyanate (clone HI30, 555482) from BD Biosciences to identify AnnV^+^ red-cell-derived, platelet-derived, or leukocyte-derived vesicles, respectively. After addition of phycoerythrin-AnnV and a cell-specific and fluorescein isothiocyanate-conjugated monoclonal antibody in AnnV buffer environment, samples were incubated in the dark for 15 min at room temperature. The samples run within 30 min in a FACScan flow cytometer (Beckton Dickinson) using CELL Quest Software (Becton Dickinson, San Jose, CA, USA). TruCount™ tubes (340334, BD Pharmingen) were used to calculate the absolute EVs count/μL.

The protein composition of EVs isolated by FFP units was estimated by immunoblotting analysis. To this purpose, EVs were precipitated with high-speed centrifugation of 1-mL FFP at 30,000× *g* for 1 h at 4°C. The produced pellet was resuspended in saline buffer and washed twice under the same conditions. The EV proteins were separated in homogeneous 10% sodium dodecyl sulfate polyacrylamide gels, and transferred onto nitrocellulose membranes. Membranes were probed with primary antibodies against vesicular proteins [anti-Hb 1:15,000, Europa Bioproducts; anti-IgGs 1:1,000 Sigma; anti-Hsp70 (K-20) 1:300 Santa Cruz Biotechnology; anti-sCLU 1:1,000 Santa Cruz Biotechnology; anti-Alix 1:1,000 Cell Signaling Technology; and monoclonal antibody against stomatin, kindly provided by Prof. R. Prohaska, Institute of Medical Biochemistry, University of Vienna, Austria] diluted in 5% non-fat milk for 1 h at room temperature. After incubation with the appropriate horseradish peroxidase-conjugated secondary antibody (1:8,000–1:14,000), the immunoreactivity was visualized by enhanced chemiluminescence.

### Statistical and Biological Network Analyses

For statistical analysis, the Statistical Package for Social Sciences (SPSS, IBM) was used. After checking all variables for normal distribution profile and presence of outliers (by using the Shapiro–Wilk test and detrended normal *Q* − *Q* plots), inter-groups differences were evaluated through independent *t*-test or Mann–Whitney test as appropriate. In addition, and according to the outcome of the normal distribution and outliers’ analyses, correlations between parameters that subsequently used for the construction of biological networks were evaluated by the Pearson’s and Spearman’s tests. The statistically significant correlations between biochemical, metabolomics, and physiological parameters collected from G6PD^−^ and control FFP samples were used for the construction of undirected biological networks. The topological representation was processed by the Cytoscape version 3.2.0 application, as previously described (6). The length of each edge was inversely proportional to the *r* value (the shortest the edge, the higher the *r* value). Significance for both network and inter-group analyses was accepted at *p* < 0.05.

### Metabolite Extraction and LC–MS Analysis

Metabolites were extracted by adding 200 µL of plasma sample (control: *n* = 5; G6PD: *n* = 6) to 200 µL of chloroform/methanol/water (1:3:1 ratio) solvent mixture stored at −20°C. Samples were vortexed for 1 min and left on ice for 2 h for complete protein precipitation. The solutions were then centrifuged for 15 min at 15,000× *g*. Twenty microliters of supernatants (two technical replicates) were injected into an ultra high-performance liquid chromatography (UHPLC) system (Ultimate 3000, Thermo) and run in positive ion mode. A Reprosil C18 column (2.0 mm × 150 mm, 2.5 µm—Dr Maisch, Germany) was used for metabolite separation. Chromatographic separations were achieved at a column temperature of 30°C and flow rate of 0.2 mL/min. A 0–100% linear gradient of solvent A (ddH2O, 0.1% formic acid) to B (acetonitrile, 0.1% formic acid) was employed over 20 min, returning to 100% A in 2 min and a 6-min post-time solvent A hold. The UHPLC system was coupled online with a mass spectrometer Q Exactive (Thermo) scanning in full MS mode (2 μscans) at 70,000 resolution in the 67–1,000 *m*/*z* range, target of 1 × 10^6^ ions, and a maximum ion injection time (IT) of 35 ms. Source ionization parameters were as follows: spray voltage, 3.8 kV; capillary temperature, 300°C; sheath gas, 40; auxiliary gas, 25; S-Lens level, 45. Calibration was performed before each analysis against positive ion mode calibration mixes (Piercenet, Thermo Fisher, Rockford, IL, USA) to ensure subppm error of the intact mass.

### Metabolomic Data Processing and Statistical Analysis

Raw files of replicates were exported and converted into mzXML format through MassMatrix (Cleveland, OH, USA), then processed by MAVEN software^1^ (21). Mass spectrometry chromatograms were elaborated for peak alignment, matching and comparison of parent and fragment ions, and tentative metabolite identification (within a 2-ppm mass-deviation range between observed and expected results against the imported KEGG database). Univariate (two-sample *t*-test, Volcano plot) and multivariate (PCA, PLS-DA) statistical analyses were performed on the entire metabolomics data set using the MetaboAnalyst 3.0 software^2^. Before the analysis, raw data were normalized by sum and pareto scaled in order to increase the importance of low-abundance ions without significant amplification of noise. False discovery rate (FDR) and Holm–Bonferroni method were used for controlling multiple testing. The web-based tools MSEA (metabolite set enrichment analysis) and MetPA (metabolic pathway analysis), which are incorporated into MetaboAnalyst platform, were used to perform metabolite enrichment and pathway analyses, respectively. Data for identified metabolites detected in all samples were submitted into MSEA and MetPA with annotation based on common chemical names. Verification of accepted metabolites was conducted manually using HMDB, KEGG, and PubChem DBs. *Homo sapiens* pathway library was used for pathway analysis. Global test was the selected pathway enrichment analysis method, whereas the node importance measure for topological analysis was the relative betweenness centrality.

## Results

### High-Antioxidant Capacity and Low-Oxidative Defects to Plasma Lipids and Proteins in FFP Units from G6PD^−^ Donors

**Figure 1:**
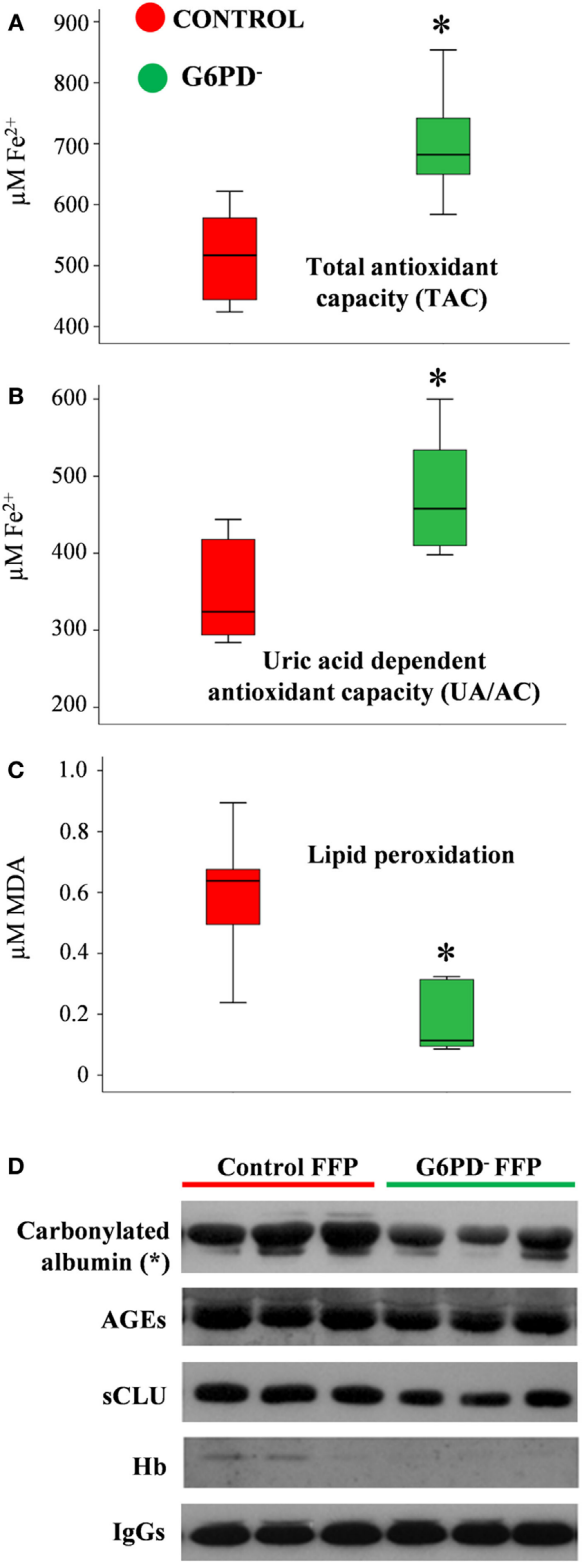
Redox status of fresh frozen plasma (FFP) units prepared from G6PD deficient (G6PD^−^) and control donors. Levels of total antioxidant capacity **(A)**, uric-acid-dependent antioxidant capacity **(B)**, and lipid peroxidation **(C)** in FFP units from G6PD^−^ donors (*n* = 6) compared with control (*n* = 6). MDA: malondialdehyde. **(D)** Representative immunoblots showing variation in the expression of stress protein markers in the two groups of FFP. AGEs: advanced glycation end-products. sCLU, soluble clusterin (apolipoprotein J). *p < 0.05, G6PD^−^ vs. control FFPs.

Oxidative stress and antioxidant capacity are donor-related factors that may contribute to the quality of FFP products. Biochemical analysis of FFP units stored for 12 months at −20°C revealed substantial differences in the oxidant/antioxidant equilibrium and the extent of oxidative defects between the two groups. Both TAC and UA/AC were significantly higher in G6PD^−^ FFP units vs. control (TAC: 698 ± 92 vs. 574 ± 52 μM Fe^2+^, UA/AC: 466 ± 89 vs. 360 ± 46 μM Fe^2+^, respectively, mean ± SD, *p* < 0.05, *n* = 12) (Figures 1A,B). In addition, the levels of lipid peroxidation, as measured by the production of malonyldialdehyde (MDA), formed during the breakdown of peroxidized fatty-acid side chains of the phospholipids (TBARS assay), was lower in the G6PD^−^ units as compared with control FFPs (0.175 ± 0.113 vs. 0.597 ± 0.219-μM MDA, *p* < 0.01, respectively, mean ± SD, *n* = 12), as shown in Figure 1C. To analyze the FFP proteins for probable oxidative defects, we performed immunoblotting analysis of an equal quantity of plasma proteins (20 µg per sample, Figure 1D). Substantially lower levels of albumin carbonylation (*p* < 0.05, *n* = 12) along with a trend for decreased levels of advanced glycation end-products (AGEs) and sCLU were detected in G6PD^−^ FFP units compared with controls. Only traces of soluble Hb were detected in some units, signifying low pre-donation levels of autohemolysis and high-quality level of the FFP preparation procedure followed, which resulted in minimal lysis of RBCs. IgG immunodetection was used for loading control.

### The EV Component of the FFP Units Differed between the Two Groups of Donors

**Figure 2:**
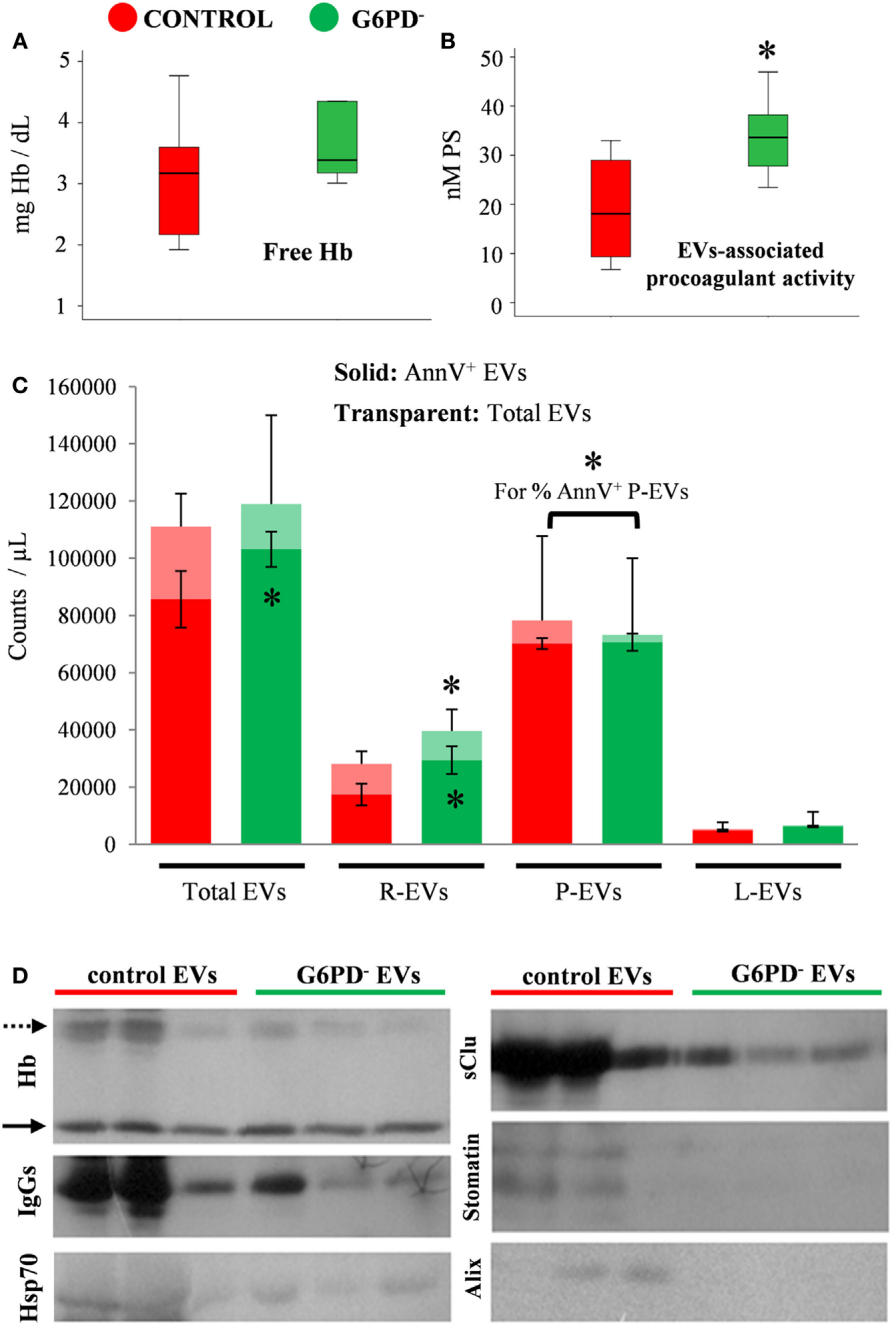
Hemolysis and extracellular vesicles (EV) analyses in fresh frozen plasma (FFP) units prepared from G6PD-deficient (G6PD^−^) and control donors. Free hemoglobin (Hb). **(A)** and EV-associated procoagulant activity **(B)** levels in the G6PD^−^ and control FFP units. **(C)** Enumeration and phenotyping of total and annexin V positive (AnnV^+^) EVs by flow cytometry. R-, P-, L-EVs stand for red cell-, platelet-, and leukocyte-derived EVs, respectively. *p < 0.05, G6PD^−^ vs. control FFPs; error bars: mean ± SD. **(D)** Representative immunoblots showing similar Hb levels (solid arrows) but variable expression of other protein components in EVs precipitated by high-speed centrifugation of equal volumes of G6PD^−^ and control FFP. Dashed arrow: oxidized Hb bands.

In large consistence with the results of the immunoblotting analysis of FFP proteins (Figure 1D), extremely low levels of free hemoglobin (<5 mg/dL) were detected by the Harboe method in all the FFP samples (*n* = 12) under study, without any inter-group difference (Figure 2A). On the contrary, the EV-associated procoagulant activity of the FFP (Figure 2B), which stands for the total concentration of PS exposed on EVs’ surface, was significantly higher in G6PD^−^ units vs. control units (33.93 ± 8.98 vs. 19.03 ± 11.76 nM PS, respectively, *p* < 0.05, mean ± SD).

To further characterize the EV part of the FFP units, we proceeded to enumeration and phenotyping analysis by flow cytometry. There was no statistically significant difference in the concentration of total EV populations between the two groups under examination; however, the concentration of AnnV^+^ EVs was higher in G6PD^−^ FFPs (Figure 2C), verifying the finding of high procoagulant activity of G6PD^−^ FFP. The platelet-derived EVs (CD41^+^) were the most abundant, followed by the red-cell-derived (CD235^+^) and the leukocyte-derived (CD45^+^) vesicles. The G6PD^−^ FFP units contained more red-cell EVs and more AnnV^+^ red-cell EVs (*p* < 0.05) compared with controls. Moreover, while similar concentrations of platelet EVs and leukocyte EVs were measured in the two FFP groups, the platelet EVs from G6PD^−^ FFP units demonstrated a higher percentage of AnnV^+^ as compared with the control group (96.5 ± 4.1 vs. 88.6 ± 5.4%, respectively, *p* < 0.05, mean ± SD).

The above-mentioned differences in the PS exposure and cell origin between the otherwise similar EV pools of G6PD^−^ and control FFPs prompted us to a rough examination of their protein composition by immunoprobing of selected components typically associated with the microvesicles or the exosomes (Figure 2D). Indeed, the vesicles precipitated by high-speed centrifugation of an equal volume (1 mL) of G6PD^−^ and control FFPs differed significantly between them in protein expression, by showing lower levels of oxidized Hb, IgGs, Hsp70, sCLU, and the red-cell lipid raft marker stomatin in the G6PD^−^ samples. As a component of the late endosomal machinery, the Alix protein has been considered a marker of endosome-derived EVs, namely of exosomes (22). Of note, traces of Alix were detected in some control samples, but not in G6PD^−^ FFP EVs.

### Metabolome FFP

**Figure 3:**
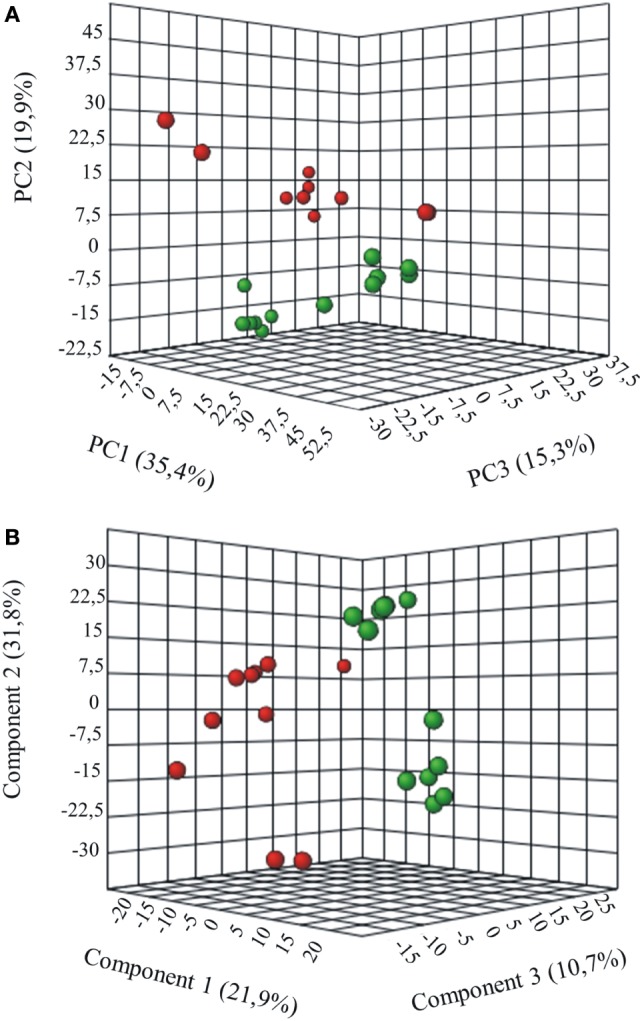
Multivariate statistical analysis of metabolomics data from control and G6PD^−^ donors. Three-dimensional principal component analysis (PCA) and partial least squares-discriminate analysis (PLS-DA) score plots are shown in **(A)** and **(B)**, respectively. Control sample groups are in red; G6PD^−^ sample groups are in green.

**Figure 4:**
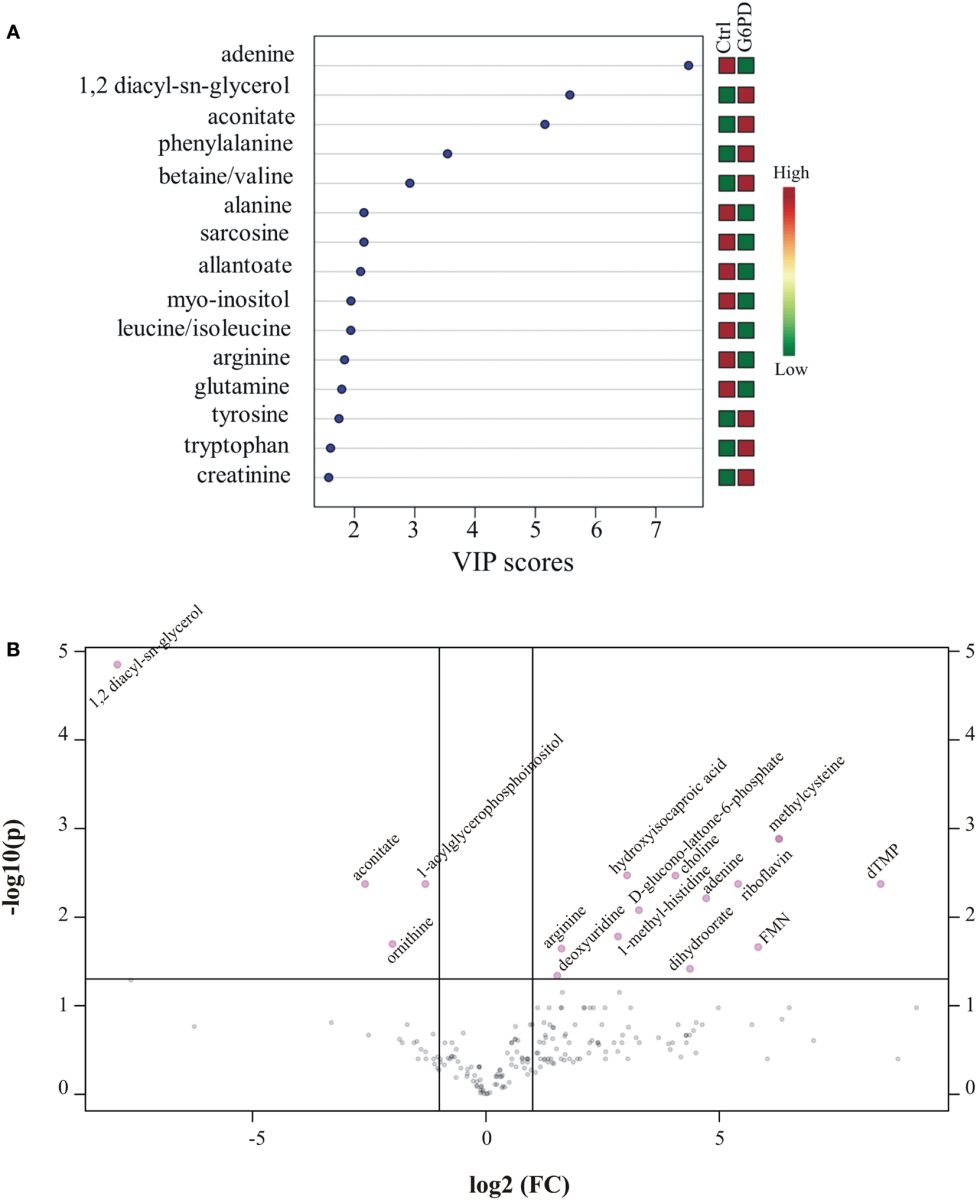
Important features identified by uni- and multivariate analyses. **(A)** G6PD^−^ vs. control variable importance in projection (VIP) plot; colored boxes on the right indicate the relative concentrations of the corresponding metabolite in each group under current study. **(B)** Volcano plot showing the distribution of the fold changes in metabolite concentrations. Metabolites with absolute fold change >2 and adjusted *p*-value (FDR < 0.05) are indicated in pink. Comparisons were analyzed using Student’s *t*-test.

**Figure 5:**
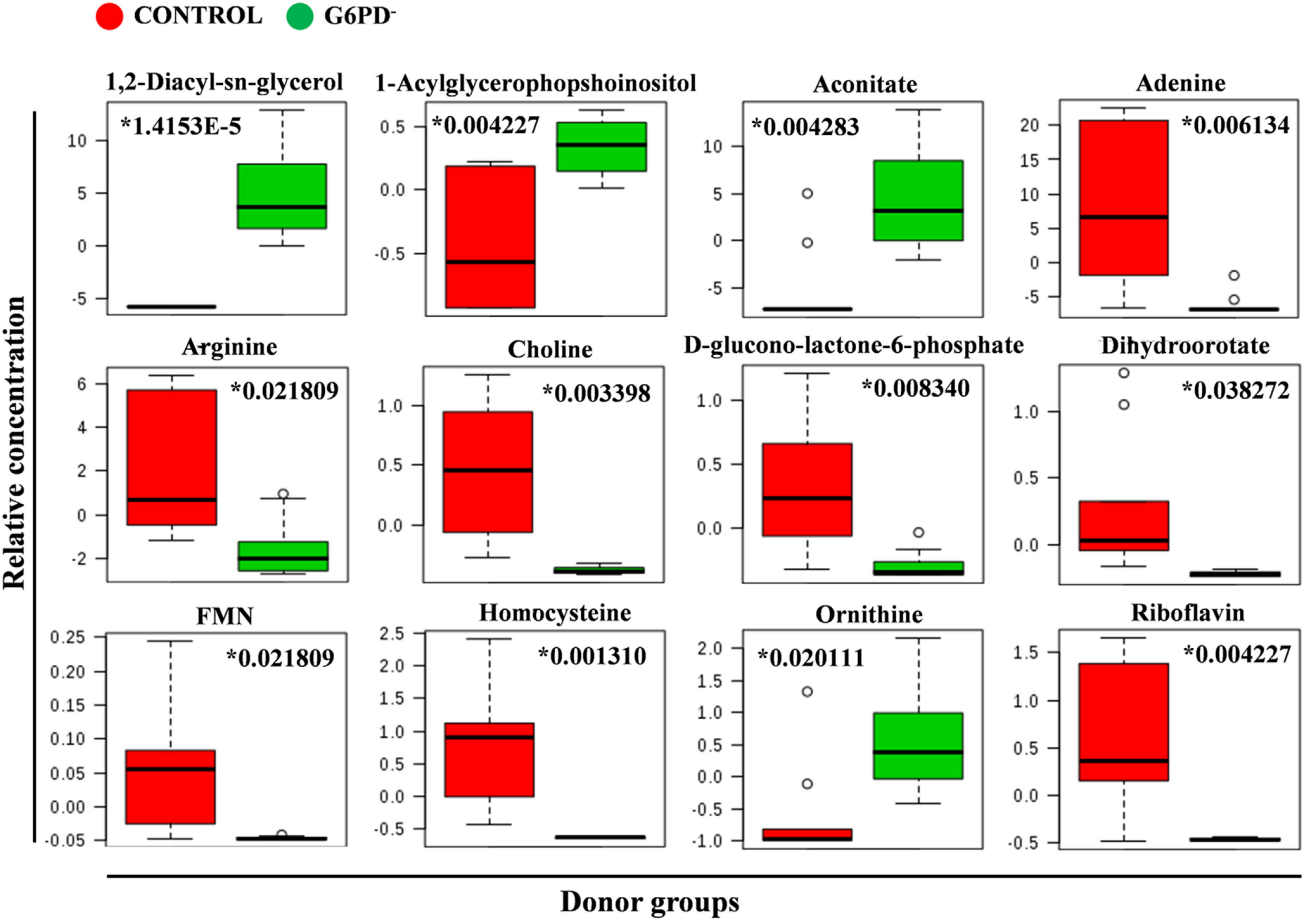
Quantitative assessment of selected metabolites. Box and whisker plots for selected significantly altered metabolites in G6PD^−^ FFP compared with control (units in normalized and scaled concentrations). The *x*-axis shows the specific metabolite and the *y*-axis is the relative concentration. Medians are indicated by horizontal lines within each box. Outliers are plotted as individual points. Numbers with asterisks indicate adjusted *p*-values.

**Figure 6:**
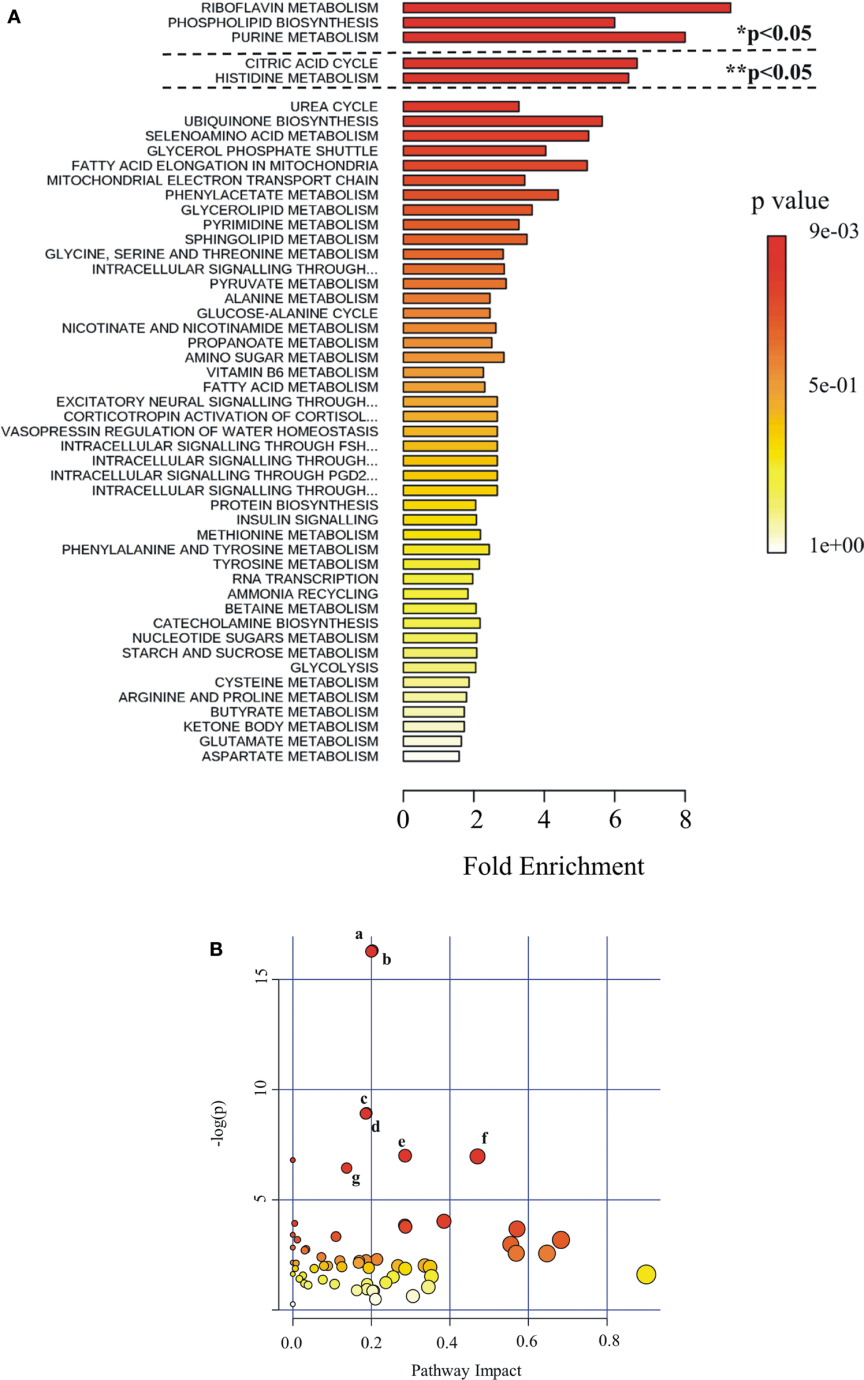
Pathway analysis as generated by MetaboAnalyst software package. Identified metabolites and their relative quantity were used to calculate the enrichment and statistical significance. **(A)** Metabolite set enrichment analysis (MSEA). Top 50 perturbed pathways are shown. The dashed lines indicate the cutoff of the adjusted *p*-value (*Holm; ** FDR). **(B)** Metabolic pathway analysis (MetPA). All the matched pathways are displayed as circles. The color and size of each circle are based on *p*-value and pathway impact value, respectively. The most impacted pathways having high statistical significance scores are indicated with letters: a, glycerolipid metabolism; b, glycerophospholipid metabolism; c, purine metabolism; d, riboflavin metabolism; e, glyoxylate and dicarboxylate metabolism; f, tricarboxylic acid cycle; g, inositol phosphate metabolism.

Metabolites were extracted from plasma samples of five healthy and six G6PD^−^ donors and were analyzed by LC–MS (two technical replicates). More than 2,000 peaks per sample were obtained referring to the KEGG database; among them, 195 metabolites were analyzed more precisely and identified. To compare the metabolomes between control and G6PD^−^ donors, both multi- and univariate statistical analyses were performed. For unsupervised multivariate analysis, principal component analysis (PCA) showed that the 70.6% of variance was captured by the first three principal components and sample groups could be clearly distinguished in the 3D-PCA score plot (Figure 3A). However, to maximize the separation achieved by PCA, partial least square discriminant analysis (PLS-DA) was subsequently performed and the obtained 3D score plots are shown in Figure 3B. The prediction accuracies were assessed by cross-validation and the best performance was obtained with three PCs (accuracy 1, R2 > 0.94, Q2 > 0.87; Figure S1 in Supplementary Material). As a supervised method, PLS-DA also enables the identification of the metabolites most contributing to the segregation of the diagnostic groups, thus variable importance in the projection (VIP) scores were calculated to rank the significance of these metabolites as potential biomarkers. Considering that variables having a VIP score of ≥1 are interpreted as being highly influential (23), 15 metabolites were considered as significant important features to differentiate control from G6PD^−^ FFP (Figure 4A). These changed plasma metabolites were mainly lipids (including 1,2-diacylglycerol, DAG), amino acids, nucleotides, and organic acids. To further confirm the specificity and significance of potentially discriminating metabolites identified from PLS-DA, univariate analysis of each metabolite was performed by combining statistical significance (Student’s *t*-test) with fold-change (FC) variations. The generated Volcano plot (FDR adjusted *p* < 0.05; FC > 2) is displayed in Figure 4B where additional metabolites included riboflavin, FMN, ornithine, D-glucono-lattone-6-phosphate, and acyl-glycerophosphoinositol, among others. Quantitative variations for a number of important metabolites are shown in Figure 5. Nevertheless, by performing PLS-DA or Volcano-plot analysis, the potential to identify subtle but substantial changes among a group of related compounds could be weakened. To overcome this obstacle, an MSEA was performed on plasma metabolites along with their relative concentrations by using the web-based platform MetaboAnalyst (Figure 6A). Metabolomic data from control and G6PD^−^ FFP showed that the pathways significantly enriched (FDR < 0.05) were as follows: (i) riboflavin metabolism, (ii) phospholipid biosynthesis, (iii) purine metabolism, (iv) tricarboxylic acid cycle, and (v) histidine metabolism. In parallel, we also utilized the MetPA module of MetaboAnalyst, which combines results from the pathway enrichment analysis with the pathway topology analysis. A graphical list of the pathways identified and their relative impact is shown in Figure 6B. The most important ones (FDR < 0.05; impact values > 0.1) included tricarboxylic acid cycle and the metabolism of the following compounds: (i) glycerolipids, (ii) glycerophospholipids, (iii) purines, (iv) riboflavin, (v) glyoxylate/dicarboxylates, and (vi) inositol phosphate. Taken together, these results point out that both analyses concurred on most of the pathways, with MetPA being slightly more sensitive.

### The Biological Networks of G6PD^−^ and Control FFP Were Different

**Figure 7:**
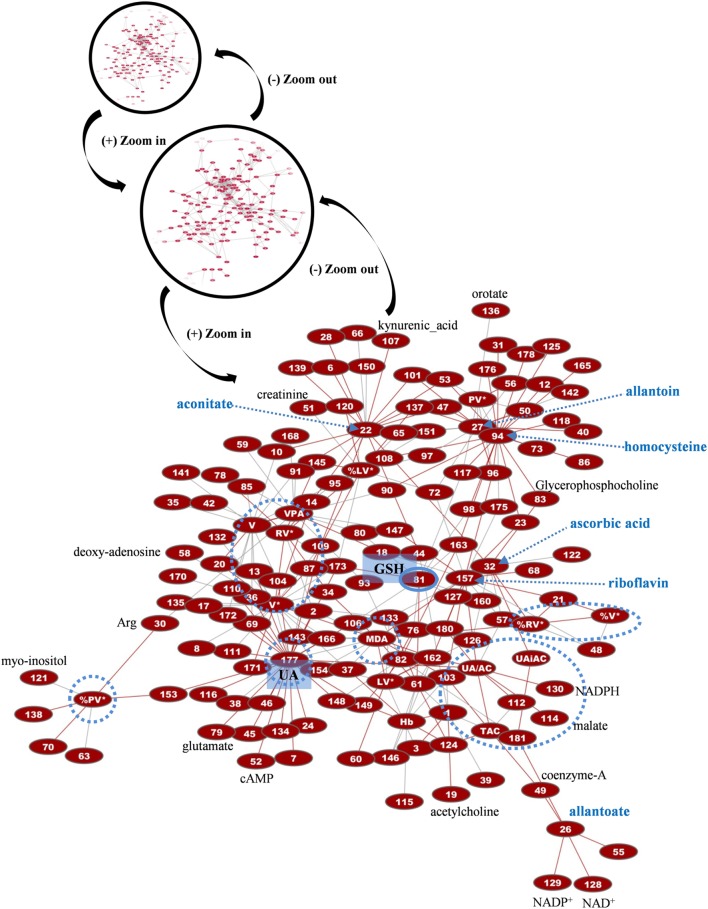
Network presentation of correlations among biochemical, physiological, and metabolomic variables in control FFP units. “Magnification” of a part of the total interactome of control fresh frozen plasma (FFP) units (networks in black circles) allowed focusing on the main connections between redox, EVs, and metabolic parameters. In the subnetwork of 266 connections (see Table S1 in Supplementary Material for code numbering and abbreviations), uric acid (UA), homocysteine, and reduced glutathione (GSH) constituted significant hub nodes, while ascorbic acid had correlations with the uric-acid-independent antioxidamt capacity of the FFP unit. Light blue dashed arrows indicate metabolites of high connectivity, and light blue circles stand for distinct groups of highly interconnected variables, or “boxes,” corresponding to FFP EVs and antioxidant capacity. Only the statistical significant correlations at *p* < 0.05 are shown. The length of each line is inversely proportional to the *r* value of the correlation (the shorter the edge, the higher the *r* value). Red lines: positive correlations; gray lines: negative correlations.

More than 1,000 statistically significant correlations (*p* < 0.05) were detected between the biochemical, physiological, and metabolic variables in control FFP units. They were topologically arranged in an untargeted biological network according to the power of the correlation coefficient *r* (the shorter the edge, the higher the *r* value, small magnification network in Figure 7). A significant part of that network (*n* = 266 pairs) referred to connections between redox, EVs, and metabolic parameters (see Table S1 in Supplementary Material for code numbering and abbreviations). Focusing on that part of the control FFP network resulted in the interactome shown in Figure 7. In that subnetwork, uric acid and uric-acid-related physiological features and metabolites (antioxidant capacity, allantoin) exhibited the higher degree of connectivity, followed by the hub nodes of EVs, homocysteine, aconitate, and riboflavin. Half of the connections involved at least one of those variables. Worth to mention here, allantoin, the precursor of allantoate in serum, is a biomarker of oxidant generation *in vivo* (24). Uric acid may be oxidized non-enzymatically to allantoin by various ROS, leading to hydrogen peroxide generation. Uric-acid-related correlations included lactate, numerous amino acids, phosphoinositol, carnitine, creatinine, and NADP/NADPH. The concentration of the AnnV^+^ EVs in the control FFP was strongly interconnected with the levels of lipid peroxidation, uric acid, adenosine, glycerophospholipids, lactate, ornithine, and, again, with several amino acids. AnnV^+^ red-cell EVs and platelet EVs had a similar degree of interconnection, while both of them, in addition to the EV-associated procoagulant activity of the FFP, strongly correlated with the levels of amino acids, lipid metabolism, and redox state components (ascorbic, uric acid, lipid peroxidation). In fact, the procoagulant activity had negative correlations with several amino acids but positive correlations with acetylcarnitine. PS exposure on platelet-derived EVs seemed to be more influenced by lipid metabolites, and thus the relevant node was arranged out of the main core of the network. Homocysteine showed significant connections with adenine and citrulline, while aconitate with citrulline, lactate, purines, choline, and ascorbic acid, among others. Riboflavin and the correlated ascorbic acid localized to the center of the network. Riboflavin had correlations with adenine, acetylcarnitine, and lipid peroxidation, while ascorbic acid with several components (adenosine-monophosphate, glycerophosphocholine, ornithine) and the uric-acid-independent antioxidant capacity of the control FFP, along with vitamin B6 metabolites.

**Figure 8:**
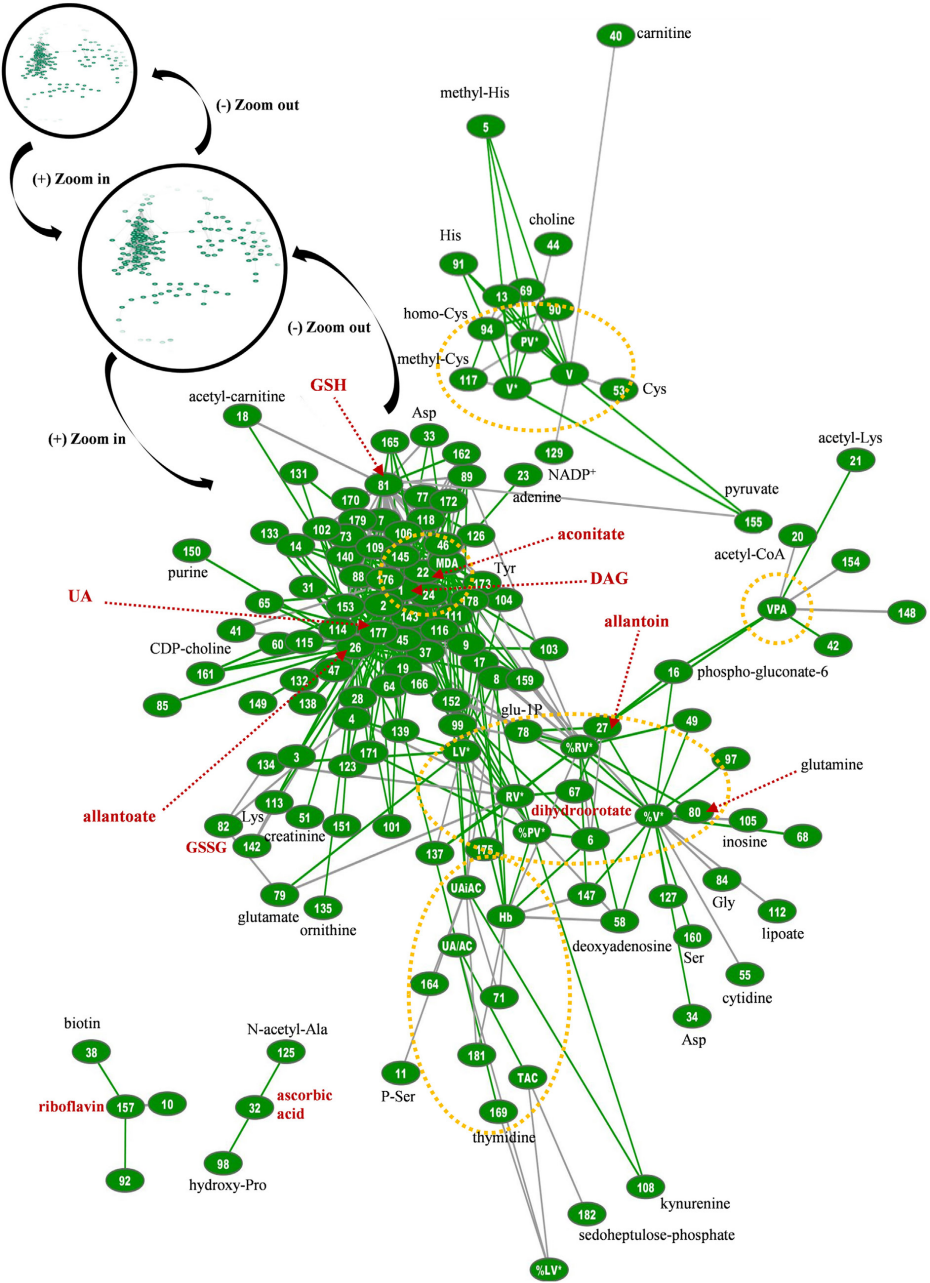
Topological presentation of correlations between biochemical, physiological and metabolomic variables in G6PD^−^ fresh frozen plasma (FFP) units. Starting from the total G6PD^−^ network (shown in black circles) and by using continuous “zoom in” tools we studied the main connections between redox, EVs, and metabolic parameters. This subnetwork was bigger and quite different compared with that of control FFP. It consisted of 434 connections (see Table S1 in Supplementary Material for code numbering and abbreviations), and while uric acid is also a hub node, diacylglycerol (DAG), lipid peroxidation (MDA), allantoate, aconitate, and reduced glutathione (GSH) have substantially more connections. The red dashed arrows indicate metabolites of high connectivity, and the yellow dashed circles stand for “boxes” of distinct groups of interconnected variables that correspond to the core of the network, FFP EVs, and antioxidant capacity. Only the statistical significant correlations at *p* < 0.05 are shown. The length of each line is inversely proportional to the *r* value of the correlation (the shorter the edge, the higher the *r* value). Green lines: positive correlations; Gray lines: negative correlations.

Regarding the biological network of G6PD^−^ FFP, it was substantially bigger than that of control FFP, with more than 2,000 statistically significant connections at total, and 434 connections in the relevant subnetwork shown in Figure 8. It was also different compared with the control network, in terms of pairing, topography, and hub nodes. While the uric-acid-related box and aconitate represented main hub nodes here too, they constructed along with the hubs of DAG, lipid peroxidation, and glutathione the extremely dense core of the network that included strongly interconnected variables. The degree of PS exposure on EVs was strongly interconnected with the levels of pyruvate, allantoin, adenosine, and inosine and, again, with several amino acids, similarly with the control network. In contrast, however, to the control FFP, the PS-exposing red-cell EVs had more connections compared with the other EV subtypes in the G6PD^−^ FFP, mostly with adenosine, amino acids, and purine metabolism variables. The platelet-EV node was again located away from the network’s core, being linked, though, with it by the pyruvate–glutathione connection. The procoagulant activity of the G6PD^−^ FFP had positive correlations with pentose phosphate pathway intermediates and allantoin. Riboflavin and ascorbic acid were not connected with the core of the network, while the uric-acid-independent antioxidant capacity had no correlation with the levels of ascorbic acid, as occurred in the control FFP. Uric acid and related variables were connected with lipid biosynthesis, transfer, and metabolism, in addition to amino acids and arginine metabolism/urea cycle components. The newly appearing hub node of DAG was strongly connected to the other hub nodes of the network, namely, the lipid peroxidation (MDA), aconitate, glutathione, and allantoate, in addition to lactate and many lipid-related, amino acids, purine, and arginine metabolism components. The hub of MDA was further connected to those of glutathione and aconitate, in addition to adenine, purine metabolism components, glycerophospholipids, and aminoacids. Glutathione hub was related to glycolysis and arginine metabolism, glycerophospholipids, and aminoacids and finally, aconitate had connections with purine and arginine metabolism, lipids, and amino acids.

## Discussion

Human plasma has been often utilized in biomarker discovery studies because its molecular composition reflects the physiological state of donor’s cells and tissues (12, 25). According to recent reports, inheritable omics variation among labile blood products, including FFP, may be associated with inter-donor differences observed in their quality, before and following transfusion (26). In similarity with the storage effect on blood components (27), G6PD^−^ affects the glycolysis and the pentose phosphate pathways, and thus, the energy and redox status of cells. Since the “fluxome,” namely, the transport properties of cell membrane, in G6PD^−^ would interconnect the intracellular and plasma metabolomes (15), we used untargeted mass spectrometry-based metabolomics strategies to study the systemic metabolic effects of G6PD^−^ on FFP used for transfusion in association with other physiological assessments, including the antioxidant capacity and the EV component, compared with G6PD^+^ controls. To the best of our knowledge, this is the first study to show FFP metabolome and physiological changes related to G6PD^−^.

### Metabolomics and Physiological Analyses Supported the Low Level of Oxidative Defects Seen in G6PD^−^ FFP Based on Uric Acid

G6PD^−^ cells are extremely sensitive to the deleterious effects of oxidants. As a probable adaptation to this inherent danger, FFP from G6PD^−^ donors was characterized by higher antioxidant capacity, which was mostly uric-acid-dependent. In fact, uric acid and its metabolic (allantoin, hypaxanthine) and physiological (antioxidant capacity) relatives constituted as high as 24% or 30% of the statistically significant correlations between the currently measured plasma variables in control and G6PD^−^ FFPs, respectively, signifying the central role of purine metabolism in plasma homeostasis. The high-antioxidant capacity of FFP in G6PD^−^ is very reminiscent of the high small molecule antioxidant capacity found in the umbilical cord blood from G6PD^−^ newborns (28). Notably, the antioxidant capacity of fresh plasma in G6PD^−^ was found similar to that of G6PD^+^ plasma (29). Thus, our data suggest either a G6PD^−^ donor variation effect or an effect of preparation and storage manipulations on the antioxidant capacity, and likely on other features of the donated plasma, which renders it only in part analogous to the *in vivo* state.

G6PD^−^ FFP under investigation had normal levels of free hemoglobin (which otherwise might trigger oxidative reactions), and substantially lower oxidative modifications to both lipids and proteins. MDA units increase as a result of oxidative stress and according to previous studies (4), their levels are influenced by pre-analytical and donor-related factors. According to our results, G6PD^−^ represents a donor-related variable with significant effects on lipid peroxidation, protein carbonylation, membrane vesiculation, and metabolome of FFP, in addition to their “wiring” in biological networks. Lipid peroxidation had significant correlations with uric acid and riboflavin in control FFP samples and with glutathione, xanthine/hypoxanthine, adenine/adenosine, and DAG in G6PD^−^ samples.

The metabolomic assessment is in harmony with the substantially low lipid peroxidation in G6PD^−^ units. Underrepresentation of riboflavin in G6PD^−^ FFP, for instance, compared with the control FFP is likely the effect of its consumption in the context of a homeostatic antioxidant activity. This water-soluble vitamin is very effective in ameliorating oxidative stress, especially lipid peroxidation, through many molecular pathways, including reduction–oxidation reactions of the molecule itself and participation in the glutathione redox cycle (30). Riboflavin is a hub node in the network of control FFP, having strong correlation with lipid peroxidation, while in G6PD^−^ FFP it correlated with glycolysis metabolites and biotin, another vitamin involved in fatty-acid metabolism and amino-acid catabolism. In addition, riboflavin exhibits anti-inflammatory and neuroprotective effects and seems to be involved in immune-mediated clinical conditions like sepsis and multiple-organ failure (30). Apart from riboflavin, the high levels of aconitate may be associated with the redox homeostasis of G6PD^−^ FFP, since in both mouse model of human G6PD^−^ (31) and in Alzheimer’s disease subjects (32) the activity of aconitase had correlations with the oxidative stress and the antioxidant protection. In our samples, aconitate was a hub node in both control and G6PD^−^ FFP networks, with significant correlations with the redox state of FFP (ascorbic acid, uric acid, lipid peroxidation), amino acids, lipids, and several metabolic pathways (glycolysis, purines, arginine). The increased antioxidant capacity but low riboflavin levels of G6PD^−^ FFP compared with control, which are reported for the first time, may be important for FFP recipients characterized by increased systemic oxidative stress, but this finding deserves further examination at clinical level.

### Increased Red-Cell EVs and EV-Associated Procoagulant Activity in G6PD^−^ FFP

By affecting the redox potential, G6PD has a critical role in the development, cell survival, and apoptotic cell death (33). PS exposure and increased vesiculation rates characterize the surface of stressed, activated, or apoptotic cells (34). Indeed, higher concentration of circulating PS^+^ microparticles (both red-cell- and platelet-derived) was reported in G6PD^−^ subjects in close association with the severity of G6PD^−^ (35). In FFP, the population of EVs is a mixture of (ex-) circulating EVs and those produced during the preparation, storage, and thawing of the unit. In our study, their cellular origin was similar to that found in previous reports (4, 13). Despite the expected (13) profound donor-dependent variation in EV enumeration among FFP samples in both groups, the G6PD^−^ samples were characterized by invariably increased procoagulant activity and percentage of PS exposure mainly on red-cell- and platelet EVs, in consistence with previous studies showing enhanced PS exposure on circulating G6PD^−^ red cells (36). Moreover, these EVs were characterized by different protein composition compared with those isolated from control FFP, and probably by a different origin, as shown by the different pattern of Alix staining. The lower expression of stress protein markers in G6PD^−^ EVs, including oxidized Hb, heat shock protein 70, and clusterin (37), was in line with the higher antioxidant capacity of the G6PD^−^ FFP.

The PS- or tissue factor-exposing EVs are likely to have procoagulant activities (38) and of note, higher levels of coagulation cascade components have been detected by proteomics analyses in EVs released by G6PD^−^ stored RBCs in comparison to controls (36). Consequently, the FFP prepared by G6PD^−^ donors probably represents a unique case, where its EV-based hemostatic activity (38–40) is combined with a high-antioxidant capacity and low-oxidative defects. The baseline rate of EV generation is strongly modulated by the endogenous or exogenous oxidative stress levels and the capacity of the antioxidant machinery, under a wide variety of physiological and pathological conditions (41). Indeed, in both FFP groups, the extent of PS exposure on EVs had significant correlations with the levels of redox state components including lipid peroxidation, uric acid/allantoin, and ascorbic acid. Moreover, the concentration of PS^+^ EVs seemed to have correlations with the levels of adenosine, amino acids, and energy metabolism, despite the fact that the individual components differed between the two groups (e.g., lactate instead of pyruvate). RBC vesiculation and EV-related procoagulant activity, which was assessed by thrombin generation *in vitro*, were found especially increased in G6PD^−^, in close association with adenosine, pentose phosphate pathway, and purine metabolism variants, showing a diverse pattern of wiring compared with the control FFP, in which ascorbic acid, lipids, and lipid modifications were more influential.

### Main Metabolic Changes in G6PD^−^ FFP with Probable Impact on Signaling

The main metabolic profile of G6PD^−^ FFP reflects to some extent a systemic cellular response to G6PD^−^, as revealed, for example, by the deficiency in gluconolactone-6-phosphate. Favic response in G6PD^−^ mice includes alterations in a similar group of plasma (e.g., ornithine) and liver (e.g., phosphoglycerols and adenine) metabolites (42). Apart from riboflavin metabolism, purine metabolism, arginine metabolism/urea cycle components, and phospholipid biosynthesis constituted the most significant differences between the G6PD^−^ and G6PD^+^ FFP units. Indeed, increased levels of ornithine were found in G6PD^−^ FFP at the expense of L-arginine, suggesting increased energy consumption/waste by the G6PD^−^ cells (43).

The significantly low extracellular levels of the purine derivative adenine in the G6PD^−^ FFP verify the previously suggested strong positive correlation of plasma adenosine (adenine nucleoside) levels with glycolysis (44, 45). RBCs, in particular, use extracellular purines to maintain their intracellular nucleotide pool and to exploit the pentose moiety for energy production. More importantly, this finding suggested a different dynamics in purinergic signaling, since purinergic receptors are widely expressed in almost every cell type, including erythrocytes (46). Adenosine arising by the metabolism of extracellular nucleotides can transmit signals through G-protein-coupled receptors and anti-inflammatory adenosine (P1 purinergic) receptors (47). Extracellular adenosine signaling serves regulatory functions in inflammation, in acute lung injury (48) and in the O_2_ delivery ability of red cell targets through boosting the production of 2,3-BPG (46). Nucleoside transporters assist in the control of plasma adenosine levels, and notably, decreased nucleoside transporter hENT1 [that also mediates hypoxanthine transport (49)] expression and activity was detected in G6PD^−^ RBCs (50). In G6PD^−^ FFP, adenosine was correlated with red-cell vesiculation, lipid peroxidation, hemolysis, DAG, oxidized glutathione, and uric acid/allantoate levels, suggesting more influential effects compared with the control FFP and a second level of intercellular signaling potential.

Aberrations in glycerolipid biosynthesis have been associated with G6PD^−^ from nematodes to humans (51). Increased activity of phospholipase A2 is observed during eryptosis which is enhanced in G6PD^−^ subjects (52). As a probable result of lower NAPDH, which is used in the reductive biosynthesis of fatty acids and cholesterol (53), lower cholesterol content (54), and clear differences in the lipid repertoire, biosynthesis and metabolism were detected between the Mediterranean type G6PD^−^ and control red cells both *in vivo* and during refrigerated storage (55). Notably, the levels of free fatty acids and oxidized derivatives were found significantly lower in stored G6PD^−^ compared with control red cells.

The presence of the highly hydrophobic DAG extracellularly was not expected. DAG has significant signaling potential that is exerted, however, at cell and subcellular membranes loci. Despite that, targeted lipidomics analysis revealed increased levels of DAG in the plasma of Alzheimer’s disease patients (56), a clinical condition with indirect, however strong, pathophysiological connections with the G6PD activity. In fact, G6PD^−^ predisposes to a variety of chronic neurological diseases by undermining the defenses against the endogenous ROS-mediated neurodegeneration during aging (57). The high oxygen concentration and fatty acids levels but low antioxidant activity in brain tissue render it highly susceptible to peroxidation and oxidative damage (58). While DAG was a minor component of the control FFP network, it was the central hub node in the G6PD^−^ network, showing numerous direct connections with glycerophospholipid biosynthesis, amino acids, and components of glycolysis, glutathione, purine, glutamate, and arginine pathways, and indirect connections with the EVs through choline, purine, and glycolysis pathways metabolites.

Partitioning of DAG into the circulating vesicles may account for its detection in biological fluids in primary G6PD^−^ and in G6PD-related clinical conditions, principally characterized by enhanced release of EVs. While exosomes are not enriched in DAG compared with the cell membrane of origin (59), DAG is a component of urinary exosomes (60, 61), and of mesenchymal stem-cell-derived EVs (62). Of note, DAG and DAG-kinase have critical role in the polarized secretion of exosomes in T and B (and probably in other kinds of) cells (63). The putative DAG-bearing exosomes in G6PD^−^ FFP may transfer powerful signaling hits to target cells through fusion or endocytosis, especially in neuronal and immune tissues that principally express the targeting molecules (namely, the C1 domain-containing proteins) (64). FFP DAG may be arisen from the hydrolysis of phosphatidic acid produced by the activity of exosomal phospholipase D (65) on extracellular phosphatidylcholine, which is quite probable, since choline—the second product of phosphatidylcholine hydrolysis by phospholipase D—was extremely low in G6PD^−^ FFP. In the same context, the levels of 1-acylglycerophosphoinositol (that is formed *via* cytidine diphosphate-DAG by reaction with inositol) were found substantially increased compared with control samples.

## Conclusion

The metabolome and several physiological features of FFP units prepared from donors with G6PD^−^ differ compared with control FFP. Higher EV-related procoagulant activity, PS concentration, red-cell vesiculation, and antioxidant capacity, along with lower oxidative modifications in lipids and proteins were detected in G6PD^−^ FFP than in G6PD^+^ units. The EVs of G6PD^−^ FFP further varied in number, cell origin, lipid and protein composition, and probably, in generation pathway. Metabolomics analysis revealed that riboflavin metabolism, purine metabolism, and glycerolipid/glycerophospholipid biosynthesis constitute the most significant variances between the two groups of FFP. Units prepared from G6PD^−^ donors had excess of DAGs, glycerophosphoinositol, aconitate, and ornithine but they were deficient in riboflavin, flavin mononucleotide, adenine, and arginine, among others. Certain FFP-needed patients may be at greatest benefit of receiving units from G6PD^−^ donors, intrinsically endowed by both procoagulant and antioxidant activities and low oxidative defects. However, the clinical outcome is likely affected by various other preparation and donor/recipient-related factors (4), including the signaling potential of the differentially expressed metabolites and EVs, the degree of G6PD^−^, the redox status in the recipient, the amount of FFP units transfused, the leukoreduction (66), and probably, the storage interval of the FFP (13). All these parameters deserve further investigation by large-scale laboratory and clinical studies.

## Ethics Statement

The study was approved by the Ethics Committee of the Department of Biology, School of Science, NKUA. Investigations were carried out upon signing of written consent in accordance with the principles of the Declaration of Helsinki.

## Author Contributions

SR and MA designed the study. VT performed the biochemical and ELISA assays. HG performed the immunoblots. AK performed the flow cytometry analysis. FG and SR performed the UHPLC-MS analyses. VT, FG, SR, and MA analyzed the results, prepared the figures, and wrote the manuscript. LZ and IP critically commented on the interpretation of data and drafting of the manuscript. All the authors contributed to the final version.

## Conflict of Interest Statement

The authors declare that the research was conducted in the absence of any commercial or financial relationships that could be construed as a potential conflict of interest.

## Footnotes

^1^http://maven.princeton.edu/.

^2^http://metpa.metabolomics.ca/.
