# Supplementary figures and images for: Redox Status, Procoagulant Activity, and Metabolome of Fresh Frozen Plasma in Glucose 6-Phosphate Dehydrogenase Deficiency
Source: Front Med (Lausanne). 2018 Feb 5;5:16. doi: 10.3389/fmed.2018.00016 (PMC5807665; doi:10.3389/fmed.2018.00016)

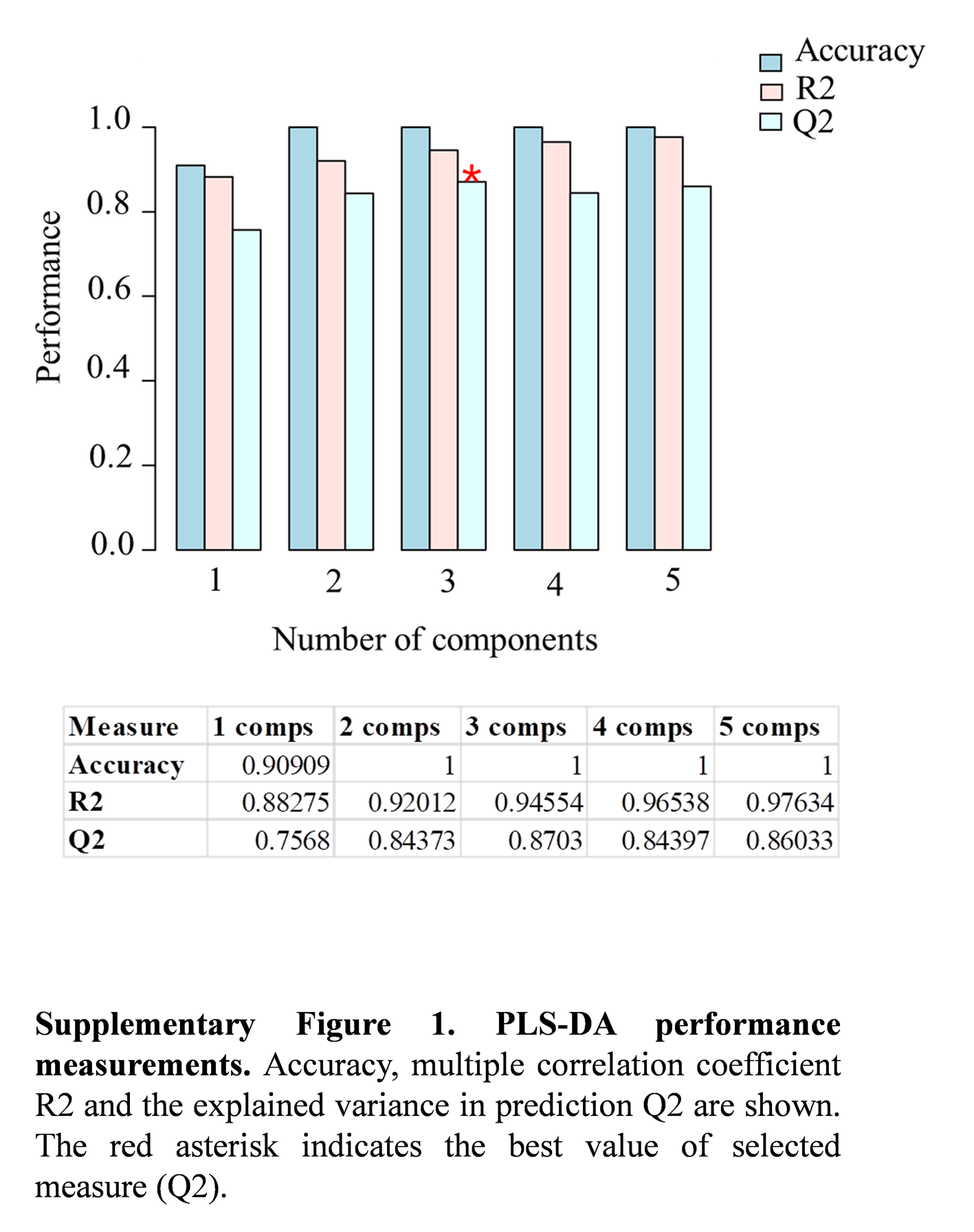

Supplement: Supplementary file 1 [file Image_1.tif]
