# Supplementary material for: Redox Status, Procoagulant Activity, and Metabolome of Fresh Frozen Plasma in Glucose 6-Phosphate Dehydrogenase Deficiency
Source: Front Med (Lausanne). 2018 Feb 5;5:16. doi: 10.3389/fmed.2018.00016 (PMC5807665; doi:10.3389/fmed.2018.00016)
Supplement: Supplementary file 2 [file Table_1.docx]

Supplementary Material

**Redox status, procoagulant activity and metabolome of fresh frozen plasma in glucose 6-phosphate dehydrogenase deficiency**

**Vassilis L. Tzounakas^1^, Federica Gevi^2^, Hara T. Georgatzakou^1^, Lello Zolla^3^, Issidora S. Papassideri^1^, Anastasios G. Kriebardis^4*^, Sara Rinalducci^2*^, Marianna H. Antonelou^1^**

^1^National and Kapodistrian University of Athens, School of Science, Department of Biology, Athens, Greece

^2^University of Tuscia, Department of Ecological and Biological Sciences, Viterbo, Italy

^3^University of Tuscia, Department of Science and Technology for Agriculture, Forestry, Nature and Energy, Viterbo, Italy

^4^Technological and Educational Institute of Athens, Faculty of Health and Caring Professions, Department of Medical Laboratories, Athens, Greece

*** Correspondence:**Anastasios Kriebardis
[akrieb@biol.uoa.gr](mailto:akrieb@biol.uoa.gr)

Sara Rinalducci

[sara.r@unitus.it](mailto:sara.r@unitus.it)

## Supplementary Table

| **SUPPL. TABLE 1** Abbreviations and annotations used in the biological networks | |
| --- | --- |
| **Parameter** | **Abbreviation** |
| **Physiological** |  |

| Concentration of PS^+^ PLT- derived EVs |  | PV* | |
| --- | --- | --- | --- |
| Concentration of PS^+^ leucocyte- derived EVs |  | LV* | |
| Concentration of PS^+^ RBC- derived EVs |  | RV* | |
| EVs-associated procoagulant activity |  | VPA | |
| Free Hb |  | Hb | |
| Malondialdhehyde |  | MDA | |
| PS^+^ total EVs |  | V* | |
| Ratio of LV*/LV |  | %LV* | |
| Ratio of PV*/PV |  | %PV* | |
| Ratio of RV*/RV |  | %RV* | |
| Total antioxidant capacity |  | TAC | |
| Total EVs |  | V | |
| UA-dependent antioxidant capacity |  | UA/AC | |
| UA-independent antioxidant capacity |  | UAiAC | |
| **Metabolites** |  |  | |
| 1-2-Diacyl-sn-glycerol | 1 | |  |
| 1-Acylglycerophosphoinositol | 2 | |  |
| 1-Acyl-sn-glycero-3-phosphocholine | 3 | |  |
| 1-Methyladenosine | 4 | |  |
| 1-Methyl-Histidine | 5 | |  |
| 2-3-dihydroxybenzoic acid | 6 | |  |
| 2-Aminooctanoic acid | 7 | |  |
| 2-oxobutanoate | 8 | |  |
| 3-methylphenylacetic acid | 9 | |  |
| 3-phosphoglycerate | 10 | |  |
| 3-phospho-serine | 11 | |  |
| 3-S-methylthiopropionate | 12 | |  |
| 4-aminobutyrate | 13 | |  |
| 4-Pyridoxic acid | 14 | |  |
| 5-methoxytryptophan | 15 | |  |
| 6-phospho-D-gluconate | 16 | |  |
| acetoacetate | 17 | |  |
| Acetylcarnitine | 18 | |  |
| Acetylcholine | 19 | |  |
| acetyl-CoA | 20 | |  |
| Acetyllysine | 21 | |  |
| aconitate | 22 | |  |
| adenine | 23 | |  |
| a-ketoglutarate | 24 | |  |
| alanine | 25 | |  |
| allantoate | 26 | |  |
| allantoin | 27 | |  |
| Aminoadipic acid | 28 | |  |
| aminoimidazole carboxamide ribonucleotide | 29 | |  |
| arginine | 30 | |  |
| anthranilate | 31 | |  |
| Ascorbic acid | 32 | |  |
| asparagine | 33 | |  |
| aspartate | 34 | |  |
| Atrolactic acid | 35 | |  |
| betaine aldehyde | 36 | |  |
| betaine/valine | 37 | |  |
| biotin | 38 | |  |
| butyryl-CoA | 39 | |  |
| carnitine | 40 | |  |
| CDP-choline | 41 | |  |
| Cholic acid | 42 | |  |
| cholesteryl sulfate | 43 | |  |
| choline | 44 | |  |
| citrate | 45 | |  |
| citrate/isocitrate | 46 | |  |
| citrulline | 47 | |  |
| CMP | 48 | |  |
| coenzyme A | 49 | |  |
| creatine | 50 | |  |
| Creatinine | 51 | |  |
| cyclic-AMP | 52 | |  |
| cysteine | 53 | |  |
| Cystine | 54 | |  |
| cytidine | 55 | |  |
| cytosine | 56 | |  |
| dAMP | 57 | |  |
| deoxyadenosine | 58 | |  |
| Deoxycholic acid | 59 | |  |
| deoxyuridine | 60 | |  |
| dephospho-CoA | 61 | |  |
| D-erythrose-4-phosphate | 62 | |  |
| D-glucarate | 63 | |  |
| D-gluconate | 64 | |  |
| D-glucono-lactone-6-phosphate | 65 | |  |
| Diacylglyceryl-N-N-N-trimethylhomoserine | 66 | |  |
| dihydroorotate | 67 | |  |
| Diiodothyronine | 68 | |  |
| dimethylglycine | 69 | |  |
| DL-Pipecolic acid | 70 | |  |
| dTDP | 71 | |  |
| dTMP | 72 | |  |
| dUMP | 73 | |  |
| FAD | 74 | |  |
| FMN | 75 | |  |
| fructose-1-6-bisphosphate | 76 | |  |
| glucosamine | 77 | |  |
| glucose-1-phosphate | 78 | |  |
| glutamate | 79 | |  |
| glutamine | 80 | |  |
| glutathione | 81 | |  |
| glutathione disulfide | 82 | |  |
| Glycerophosphocholine | 83 | |  |
| glycine | 84 | |  |
| glycolate | 85 | |  |
| glyoxylate | 86 | |  |
| GMP | 87 | |  |
| Guanidoacetic acid | 88 | |  |
| guanine | 89 | |  |
| guanosine | 90 | |  |
| histidine | 91 | |  |
| histidinol | 92 | |  |
| homocysteic acid | 93 | |  |
| homocysteine | 94 | |  |
| homoserine | 95 | |  |
| Hydroxyisocaproic acid | 96 | |  |
| hydroxyphenylpyruvate | 97 | |  |
| hydroxyproline | 98 | |  |
| hypoxanthine | 99 | |  |
| IDP | 100 | |  |
| Imidazoleacetic acid | 101 | |  |
| indole | 102 | |  |
| Indole-3-carboxylic acid | 103 | |  |
| Indoleacrylic acid | 104 | |  |
| inosine | 105 | |  |
| isocitrate | 106 | |  |
| Kynurenic acid | 107 | |  |
| Kynurenine | 108 | |  |
| lactate | 109 | |  |
| L-arginino-succinate | 110 | |  |
| leucine/isoleucine | 111 | |  |
| lipoate | 112 | |  |
| lysine | 113 | |  |
| malate | 114 | |  |
| malonyl-CoA | 115 | |  |
| methionine | 116 | |  |
| Methylcysteine | 117 | |  |
| Methylmalonic acid | 118 | |  |
| methylnicotinamide | 119 | |  |
| Methionine sulfoxide | 120 | |  |
| myo-inositol | 121 | |  |
| N-acetyl-glucosamine-1/6-phosphate | 122 | |  |
| N-acetyl-glutamate | 123 | |  |
| N-acetyl-glutamine | 124 | |  |
| N-Acetyl-L-alanine | 125 | |  |
| N-acetyl-L-ornithine | 126 | |  |
| N-Acetylputrescine | 127 | |  |
| NAD+ | 128 | |  |
| NADP+ | 129 | |  |
| NADPH | 130 | |  |
| NG-dimethyl-L-arginine | 131 | |  |
| nicotinamide | 132 | |  |
| nicotinate | 133 | |  |
| O-acetyl-L-serine | 134 | |  |
| ornithine | 135 | |  |
| orotate | 136 | |  |
| oxaloacetate | 137 | |  |
| p-aminobenzoate | 138 | |  |
| pantothenate | 139 | |  |
| phenylalanine | 140 | |  |
| Phenyllactic acid | 141 | |  |
| Phenylpropiolic acid | 142 | |  |
| phenylpyruvate | 143 | |  |
| Phosphatidylglycerol | 144 | |  |
| phosphoenolpyruvate | 145 | |  |
| Phosphorylcholine | 146 | |  |
| p-hydroxybenzoate | 147 | |  |
| prephenate | 148 | |  |
| proline | 149 | |  |
| purine | 150 | |  |
| Pyridoxamine | 151 | |  |
| pyridoxine | 152 | |  |
| Pyroglutamic acid | 153 | |  |
| Pyrophosphate | 154 | |  |
| pyruvate | 155 | |  |
| quinolinate | 156 | |  |
| riboflavin | 157 | |  |
| sarcosine | 158 | |  |
| Sedoheptoluse bisphosphate | 159 | |  |
| serine | 160 | |  |
| S-methyl-5--thioadenosine | 161 | |  |
| sn-glycero-3-Phosphocholine | 162 | |  |
| sn-glycerol-3-phosphate | 163 | |  |
| S-ribosyl-L-homocysteine-nega | 164 | |  |
| succinate | 165 | |  |
| taurine | 166 | |  |
| thiamine | 167 | |  |
| threonine | 168 | |  |
| thymidine | 169 | |  |
| thymine | 170 | |  |
| trehalose/sucrose | 171 | |  |
| tryptophan | 172 | |  |
| tyrosine | 173 | |  |
| UDP | 174 | |  |
| UDP-D-glucose | 175 | |  |
| uracil | 176 | |  |
| Uric acid | 177 | |  |
| uridine | 178 | |  |
| xanthine | 179 | |  |
| xanthosine | 180 | |  |
| Xanthurenic acid | 181 | |  |
| D-sedoheptulose-1-7-phosphate | 182 | |  |

## Legend for the Supplementary Figure

**Supplementary Figure 1. PLS-DA performance measurements.** Accuracy, multiple correlation coefficient R2 and the explained variance in prediction Q2 are shown. The red asterisk indicates the best value of selected measure (Q2).

**
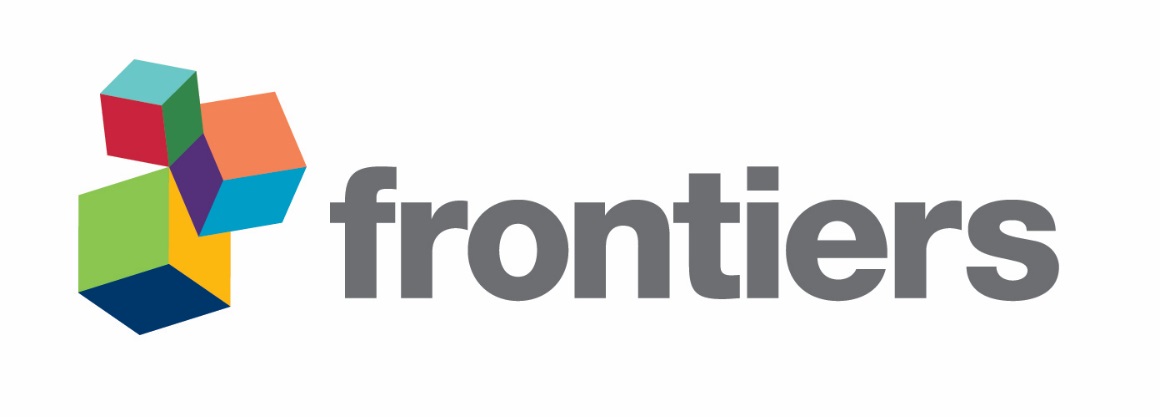
**
